# Meditating on psychedelics. A randomized placebo-controlled study of DMT and harmine in a mindfulness retreat

**DOI:** 10.1177/02698811241282637

**Published:** 2024-09-27

**Authors:** Daniel Meling, Klemens Egger, Helena D Aicher, Javier Jareño Redondo, Jovin Mueller, Joëlle Dornbierer, Elijah Temperli, Emilia A Vasella, Luzia Caflisch, David J Pfeiffer, Jonas TT Schlomberg, John W Smallridge, Dario A Dornbierer, Milan Scheidegger

**Affiliations:** 1 Psychedelic Research and Therapy Development, Department of Adult Psychiatry and Psychotherapy, Psychiatric University Clinic Zurich, University of Zurich, Zurich, Switzerland; 2 Department of Psychosomatic Medicine and Psychotherapy, Medical Center—University of Freiburg, Faculty of Medicine, University of Freiburg, Freiburg, Germany; 3 Neuroscience Center Zurich, Swiss Federal Institute of Technology Zurich, University of Zurich, Zurich, Switzerland; 4 Department of Nuclear Medicine, Bern University Hospital, Bern, Switzerland; 5 Department of Psychology, University of Zurich, Zurich, Switzerland; 6 Digital Society Initiative, University of Zurich, Zurich, Switzerland

**Keywords:** Psychedelic, meditation, mindfulness, DMT, dimethyltryptamine, ayahuasca, mystical experience, insight, compassion

## Abstract

**Background:**

In recent years, both meditation and psychedelics have attracted rapidly increasing scientific interest. While the current state of evidence suggests the promising potential of psychedelics, such as psilocybin, to enhance meditative training, it remains equivocal whether these effects are specifically bound to psilocybin or if other classical psychedelics might show synergistic effects with meditation practice. One particularly promising candidate is *N,N*-dimethyltryptamine (DMT), an active ingredient of ayahuasca.

**Aim:**

This study aims to investigate the effect of the psychedelic substance DMT, combined with the monoamine oxidase inhibitor harmine (*DMT-harmine*), on meditative states, compared to meditation with a placebo.

**Method:**

Forty experienced meditators (18 females and 22 males) participated in a double-blind, placebo-controlled study over a 3-day meditation retreat, receiving either placebo or DMT-harmine. Participants’ levels of mindfulness, compassion, insight, and transcendence were assessed before, during, and after the meditation group retreat, using psychometric questionnaires.

**Results:**

Compared to meditation with a placebo, meditators who received DMT and harmine self-attributed greater levels of mystical-type experiences, non-dual awareness, and emotional breakthrough during the acute substance effects and, when corrected for baseline differences, greater psychological insight 1 day later. Mindfulness and compassion were not significantly different in the DMT-harmine group compared to placebo. At 1-month follow-up, the meditators who received DMT and harmine rated their experience as significantly more personally meaningful, spiritually significant, and well-being-enhancing than the meditators who received placebo.

**Conclusion:**

Investigating the impact of DMT-harmine on meditators in a naturalistic mindfulness group retreat, this placebo-controlled study highlights the specific effects of psychedelics during meditation.

**Trial registration:**

ClinicalTrials.gov identifier NCT05780216.

## Introduction

Meditation and psychedelics have attracted growing scientific interest over the past few years (Davidson, 2021; Hadar et al., 2022; Lawrence et al., 2021; Van Dam et al., 2018), as reflected in a large increase in the number of scientific publications on meditation (Van Dam et al., 2018; Williams and Kabat-Zinn, 2011) and psychedelics (Hadar et al., 2022; Petranker et al., 2020).

Meditation can be defined as practices that promote human flourishing by training the mind (Dahl and Davidson, 2019). These practices can be distinguished in relation to four core dimensions of well-being, including (1) awareness, (2) connection, (3) insight, and (4) purpose (Dahl et al., 2020). Thereby, meditation can be understood as a form of mental training with various aims, among them improving attentional and emotional self-regulation, perspective-taking and cognitive reappraisal, and generating insights into one’s notion of the self, others, and the world (Dahl et al., 2015; Tang et al., 2015).

Psychedelics, on the other hand, are a broad class of psychoactive substances that induce altered states of consciousness, including alterations in perception, cognition, emotion, and sense of self (Carhart-Harris et al., 2016a, 2016b; Griffiths et al., 2006, 2016; Muttoni et al., 2019).

In recent years, research on meditation and psychedelics has brought forth growing evidence of their respective benefits for mental health and well-being. Meditation has been shown to improve mental health and psychological well-being across a multitude of randomized controlled studies (Goldberg et al., 2018; Rose et al., 2020). Similarly, expanding research on psychedelics provides growing evidence of their promising therapeutic potential for treating a broad range of mental health conditions (Andersen et al., 2021; Goldberg et al., 2020; Hadar et al., 2022; Lawrence et al., 2021; Romeo et al., 2020).

While the respective therapeutic evidence for meditation and psychedelics has been established as standalone interventions, recent research has started to point at their similarities (Millière et al., 2018; Scheidegger, 2021), differences (Heuschkel and Kuypers, 2020), and linkages (Simonsson et al., 2023; Simonsson and Goldberg, 2023), culminating in pointers toward potential synergies in combining psychedelics and meditation practice (Eleftheriou and Thomas, 2021; Griffiths et al., 2018; Payne et al., 2021; Smigielski et al., 2019a).

In pursuit of understanding the potential synergies between meditation and psychedelics, recent randomized controlled trials have yielded intriguing results. Studies investigating the use of psilocybin within mindfulness retreats or meditation programs have demonstrated notable increases in both the frequency and depth of meditation practice. Moreover, psilocybin was shown to induce profound states of self-transcendence that were linked to larger positive follow-up changes in behavior and attitudes (Griffiths et al., 2018; Smigielski et al., 2019a). At 6 months of follow-up, participants in the study by Griffiths et al. (2018) showed increased interpersonal closeness, gratitude, sense of purpose and life meaning, forgiveness, transcendence of death, and daily spiritual experiences. Interestingly, the determinants of these effects were predicted by both the intensity of the psilocybin-occasioned mystical experience and the degree of engagement with meditative practices (Griffiths et al., 2018). Smigielski et al. (2019a) found that experiences of unity and blissfulness, spiritual experiences, and insightfulness (all captured as subscales of the 5D-ASC questionnaire) were significantly higher in meditators who received psilocybin compared to those who received placebo. Moreover, the magnitude of the mystical experience as well as corresponding changes in brain connectivity predicted positive changes in participants’ psychosocial behavior and attitudes at the 4-month follow-up (Smigielski et al., 2019b). On the neurobiological level, the combination of psilocybin and meditation showed modulations in self-referential networks including changes in the brain’s default-mode network connectivity (Smigielski et al., 2019b). Moreover, it showed an increased optimal transport distance (i.e., increased dissimilarity and decreased overlap) particularly between open monitoring meditation and resting state, using a novel *Mapper* approach based on topological data analysis (Singer et al., 2024). These findings suggest critical experiential and neurobiological differences between meditation with psilocybin versus meditation alone.

Besides the differences between meditation with psilocybin and meditation alone, there are also differences between psilocybin with meditation versus psilocybin alone. Compared to non-meditators who received the same dose of psilocybin in a different study (Studerus et al., 2012), the combination of psilocybin and meditation resulted in significantly higher ratings of unity (70% vs 40%), blissfulness (86% vs 48%), and spiritual experience (66% vs 22%), and also lower ratings of anxious ego dissolution, and vigilance reduction while auditory and visual alterations did not differ (Smigielski et al., 2019a; Studerus et al., 2012).

Moreover, the first studies on potential synergies between meditation and psychedelics suggest combined effects toward enhanced (1) mindfulness, (2) connectedness and potentially compassion, (3) insight, and (4) transcendent mystical-type experiences.

First, previous research showed that psilocybin-enhanced meditation increased post-intervention mindfulness to a larger degree than meditation with a placebo (Smigielski et al., 2019a), suggesting a promising potential of psychedelics to augment mindfulness training.

Second, while a qualitative study on the clinical use of psilocybin showed that all 17 patients referred to the sense of connectedness as a common factor for the acute and enduring effects of psychedelics (Watts et al., 2017), it has been argued that psychedelic-assisted psychotherapy targets the sense of connectedness as a common factor (Carhart-Harris et al., 2018). Another qualitative study in the clinical domain reported that, besides the feelings of interconnectedness (8 out of 13 participants), participants reported that psilocybin also supported acute states of self-compassion (9 out of 13 participants; Agin-Liebes et al., 2023). As a potential synergy between meditation and psychedelics, Payne et al. (2021) have argued that psychedelics may support meditation practice by eliciting a type of mindfulness that is kinder and more compassionate in contrast to mindfulness that is more rigid, self-centered, or controlling.

Third, previous research found that insightfulness was significantly increased in psilocybin-enhanced meditation, compared to meditation with a placebo (Smigielski et al., 2019a). Moreover, clinical evidence suggests that psychedelic-occasioned insight experiences predict positive outcomes of psychedelic therapy (Davis et al., 2020; Garcia-Romeu et al., 2019, 2020). A survey study with 2822 respondents in the United States showed that these psychedelic-occasioned insights were associated with increased engagement in current meditation practice, including mindfulness and compassion meditation (Simonsson et al., 2023).

Fourth, it has been repeatedly shown that psilocybin can occasion mystical-type experiences in combination with meditation (Griffiths et al., 2018; Smigielski et al., 2019a) as well as in other contexts (Griffiths et al., 2006, 2008, 2011, 2016). Furthermore, psilocybin-induced states of transcendence were linked to larger positive follow-up changes in attitudes and behavior (Griffiths et al., 2018; Smigielski et al., 2019a).

Taken together, this current state of evidence suggests the potential of psychedelics to enhance mindfulness, connectedness, and potentially compassion, insight, and mystical-type transcendence. However, it remains unclear whether the presented effects are specific to psilocybin or if other classic psychedelic substances are equally compatible with meditation practice and reliable enhancers of mindfulness, compassion, insight, and transcendence.

One particularly promising candidate in this regard is *N,N*-dimethyltryptamine (DMT). DMT is a psychoactive component that is found in many plants across the globe (e.g., Acacia family, Mimosa family, reeds, grasses), some of which are recognized as medicinal plants and used for the Indigenous Amazonian plant medicine ayahuasca. Ayahuasca has recently attracted increasing scientific interest with growing evidence of its potential therapeutic effects, including reducing symptoms of anxiety and depression (dos Santos et al., 2016; Palhano-Fontes et al., 2019; Osório et al., 2015; Sarris et al., 2021). Moreover, it was shown that ayahuasca may enhance mindfulness-related capabilities, such as decentering, non-reactivity, and acceptance (Sampedro et al., 2017; Soler et al., 2016) and increase empathy (Kiraga et al., 2021) and self-compassion (Domínguez-Clavé et al., 2022).

Besides DMT, ayahuasca contains a variety of β-carboline alkaloids including harmine, harmaline, and tetrahydroharmine (THH) which act as selective reversible monoamine oxidase (MAO) inhibitors that prevent the fast degradation of DMT in the body (Callaway et al., 1996; Egger et al., 2024). At the administered doses, harmine primarily acts as an MAO inhibitor and pharmacokinetic enhancer of DMT. However, it also strongly binds to DYRK1A, shows moderate affinity for 5-HT2A and imidazoline I2 receptors, and has some affinity for 5-HT2C receptors and the dopamine transporter (Brierley and Davidson, 2012). Previous studies showed that isolating DMT and harmine and bypassing the gastrointestinal (GI) tract through administering harmine buccally and DMT intranasally (among other possibilities) showed a substantial reduction in GI-related side effects including nausea, vomiting, and diarrhea (Dornbierer et al., 2023). On the other hand, therapeutically relevant psychedelic and empathogenic effects, including phenomenologically rich psychedelic experiences, psychological insights, and emotional breakthroughs could be maintained (Aicher and Mueller et al., 2024). This combination of DMT and harmine or other MAO inhibitors is commonly referred to as an “ayahuasca analog” or “ayahuasca-inspired DMT-harmine formulation” (Aicher and Mueller et al., 2024). Throughout this article, the term “*DMT-harmine*” will be used.

Importantly, previous basic research in laboratory settings showed that DMT-harmine enhanced mindfulness, self-compassion, and compassion for others for up to 24 h after intake in healthy participants. These effects from a moderate dose of DMT-harmine were short term and did not sustain at a 1-month or 4-month follow-up (Aicher and Wicki et al., personal communication). These findings, however, were collected in non-meditators in a context that did not emphasize mindfulness or compassion. Therefore, this leaves the question open whether pronounced and sustained increases in mindfulness and compassion are more likely in meditators and when DMT-harmine is administered in a mindfulness and compassion meditation retreat.

Taken together, this current state of evidence suggests the potential of ayahuasca and ayahuasca-inspired DMT-harmine formulations to support capabilities related to mindfulness, compassion, and psychological insight. However, it remains unclear whether these effects go beyond the effects of meditation alone and whether these effects are even further increased in combination with meditation.

Given the presented current state of research on psilocybin combined with meditation on the one hand and ayahuasca or DMT-harmine without meditation on the other hand, we hypothesize that DMT-harmine combined with meditation increases (1) mindfulness, (2) compassion, (3) insight, and (4) mystical-type transcendence to a larger degree than meditation with a placebo.

## Methods

### Participants

In all, 40 intermediate meditation practitioners were recruited via announcements on the Internet and e-mail newsletters of local meditation communities. For logistical reasons, two separate meditation retreats were held, with 19 and 21 participants, respectively. The study was advertised as investigating the effects of meditation and DMT combined with harmine. A total of 102 people applied for participation, of which 84 were screened by telephone and 44 were further screened in person, including a medical check-up and psychological assessment. The 41 qualified study participants were psychiatrically and medically healthy (according to medical history, physical examination, an electrocardiogram, routine blood analysis, and urine tests for common drugs of abuse). As assessed through structured clinical interviews, participants were without a personal history of bipolar disorder (type I or II), schizophrenia, schizoaffective disorder, psychosis, or other disorders from the psychotic spectrum. Also, individuals with family histories of schizophrenia, schizoaffective disorder, or bipolar disorder type I were excluded. Individuals with current abuse of medication or psychotropic substances (including nicotine addiction) were excluded. Female participants capable of bearing children additionally completed a urine pregnancy test to rule out pregnancy during study participation. One participant of the first retreat withdrew their participation due to health reasons, which was compensated for in the second retreat, leading to a total of 40 participants (18 females and 22 males) completing the study.

Participants had a mean age of 43.73 years (27–60, SD: 10.30) and 82.5% had postgraduate degrees. 95% of the participants were White. Participants reported an average of 2422.5 h of meditation (800–10,000), coming from various traditions, including Zen, Vipassana, and Tibetan Buddhism.

The study was approved by the Cantonal Ethics Committee of Zurich, Switzerland, and received an exemption from the Federal Office of Public Health (FOPH) for the administration of the controlled substance DMT. Written informed consent was obtained from all participants. The study was performed according to the Declaration of Helsinki (ClinicalTrials.gov identifier: NCT05780216).

### Study procedures and setting

The study followed a double-blind, placebo-controlled, between-subject design and investigated the effects of DMT-harmine on multiple attitudinal and experiential outcome measures.

Each of the 40 participants was randomly assigned to one of two groups (placebo, DMT-harmine) which were matched for gender. The two groups were similar across a variety of baseline variables, including age and lifetime meditation hours (see Table S1, Supplemental Material). Participants completed one of two subsequent 3-day meditation retreats, with 19 participants (9 DMT-harmine, 10 placebo) in the first retreat and 21 participants (11 DMT-harmine, 10 placebo) in the second retreat.

The 3-day retreats took place at Felsentor in Switzerland, a Zen meditation center where participants practiced mindfulness and compassion meditation in structured daily group sessions. Each day of the retreat, sitting meditation in the zendo, that is, a traditional Zen meditation hall, was interleaved with walking meditation, mindful physical work, short breaks, and meals.

On the second day of the retreat, participants continued their meditation practice while receiving incremental doses of DMT-harmine or placebo, administered in a double-blind manner (see section “Substance and dosing”). The dose administrations started at 10:30 in the morning followed by periods of (1) sitting meditation (20 min), (2) vital sign measurements and acute psychometry (5 min), and (3) walking meditation (5 min). Placebo or DMT-harmine was administered after the walking meditation and before the next sitting meditation period. Moreover, the adapted retreat schedule contained additional musical elements and relaxation periods after the last dose administration (see section “Substance and dosing”). Participants were encouraged to engage in a formal meditation posture only to the extent to which it remained comfortable to them and to take a gentle approach to meditation with an effortless rather than forceful attitude.

Accordingly, the retreat consisted of three phases: preparation (day 1), placebo or DMT-harmine administration (day 2), and integration (day 3). During the retreat, questionnaires were filled out at the end of day 1 (18:30), day 2 (14:30 and 16:30), and day 3 (18:30). On day 2, acute psychometric questions were filled out at baseline as well as 30, 60, 90, 120, 180, 240, and 360 min after the first drug/placebo intake. Moreover, participants filled out questionnaires 1 day before and 1 day after the retreat, as well as at 1-week follow-up and 1-month follow-up. See the section “Outcome measures” for a more detailed description of the questionnaires and their respective time points.

### Substance and dosing

The pharmacological intervention utilized a standardized sublingual formulation containing DMT hemisuccinate and harmine glucuronate. DMT was extracted from the plant Mimosa hostilis and purified through vacuum filtration, organic extraction with n-heptane, and recrystallization according to well-established procedures (see Dornbierer et al., 2023). Harmine, sourced from Santa Cruz Biotechnology (Dallas, Texas, USA), underwent further purification via acidic aqueous extraction, formation of its hydrochloride salt, and precipitation of the freebase. The sublingual formulation was made by blending the active drugs with calcium phosphate, sucralose, and flavors for taste masking (menthol, peppermint). The blend was then compacted into fast-disintegrating orodispersible tablets using TIP (template inverted particles). The tablets contained 30 mg of DMT and 30 mg of harmine (both as freebase weight), and were administered in four increments every 30 min, resulting in a total dosage of 120 mg per session. One dose of DMT-harmine tablets provided an equivalent amount of DMT to an average dose of ayahuasca. However, the harmine dose was lower than the typical amount of MAO inhibitors found in ayahuasca, ensuring a safe dosage regimen compatible with meditative practice. The placebo tablets matched the verum tablets in appearance and taste.

### Baseline measures

To screen the study sample, the following data were collected at baseline: demographic data, lifetime hours of formal meditation, history of drug use, and brief symptom checklist.

### Outcome measures

The central outcome measure of this study can be distinguished into four clusters: (1) mindfulness, (2) compassion, (3) insight, and (4) transcendence. Additional outcome measures included items on the perceived meaningfulness and spiritual significance of the study experience and items assessing the blinding efficacy of the double-blind study design. An overview of the measurement time points for each questionnaire is provided in Table 1.

**Table 1.** Measurement time points for each of the reported questionnaires.

Construct	Questionnaire	Baseline	Day 1	Day 2	Day 3	Post	1-week follow-up	1-month follow-up
**Mindfulness**	TMS-13		x	x	x			
	FMI-13	x				x	x	
**Compassion**	SOCS-O	x		x		x	x	
	SOCS-S	x		x		x	x	
**Insight**	PIS-6		x	x	x			
	EBI		x	x	x			
**Transcendence**	MEQ30		x	x	x			
	NADA-S		x	x	x			
**Blinding efficacy**				x				
**Meaningfulness**								x

TMS-13: Toronto Mindfulness Scale-state version; FMI-13: Freiburg mindfulness inventory-13; SOCS-O and -S: Sussex-Oxford compassion scales for self and other; PIS-6: psychological insight scale; EBI: emotional breakthrough inventory; MEQ30: mystical experience questionnaire; NADA-S: nondual awareness dimensional assessment—state; Baseline: 1 day before the retreat; Post: 1 day after the retreat.

#### Mindfulness

State mindfulness was measured on each day of the retreat using the Toronto Mindfulness Scale (TMS; Lau et al., 2006). The TMS-13 includes 13 items and is specifically state oriented for use immediately following a meditation practice session. This instrument consists of two factors, *curiosity* and *decentering*. The items of the first factor (curiosity) are designed to capture an attitude in which one is oriented toward learning more about one’s subjective experiences. The items of the second factor (decentering) are designed to capture to which extent a person identifies with experiences or rather sees them as mere thoughts and emotions.

Trait mindfulness, as measured by the Freiburg Mindfulness Inventory (FMI-13; Walach et al., 2006), was evaluated the day before the retreat, the day after the retreat, and 1 week after the retreat. The FMI-13 is a 13-item questionnaire capturing a one-dimensional construct of mindfulness derived from Buddhist psychology.

#### Compassion

Compassion was evaluated using the Sussex-Oxford Compassion Scales (SOCS; Gu et al., 2020). The SOCS includes the 20-item Sussex-Oxford Compassion for Others Scale (SOCS-O) and the 20-item Sussex-Oxford Compassion for the Self Scale (SOCS-S). The SOCS-O and SOCS-S are based on a definition of compassion that includes five dimensions: (1) recognizing suffering, (2) acknowledging the universality of suffering, (3) feeling for the person suffering, (4) tolerating unpleasant experiences, and (5) motivation and determination to act toward alleviating suffering.

#### Insight

Insightfulness was evaluated using the Psychological Insight Scale (PIS-6; Peill et al., 2022). The PIS-6 consists of six items that are designed to capture the level of psychological insight, specifically after a psychedelic experience.

Often accompanied by personal and transpersonal insights and related to psychological insights, emotional breakthroughs were captured, using the Emotional Breakthrough Inventory (EBI; Roseman et al., 2019). It consists of six items that are used to evaluate an emotional insight quality, which is typical for psychedelic experiences.

#### Transcendence

Mystical-type experiences were evaluated using the Mystical Experience Questionnaire (MEQ30; Barrett et al., 2015). The MEQ30 consists of 30 items to capture individual mystical experiences. The MEQ30 has been shown to predict sustainable changes in attitudes, behavior, and well-being associated with psilocybin-occasioned experiences (Barrett et al., 2015).

To assess the immediate effect of meditation practice on non-dual awareness, we used the Nondual Awareness Dimensional Assessment-State (NADA-S; Hanley et al., 2018). The NADA-S consists of three items, which are designed to measure fluctuations in non-dual states of awareness evoked through contemplative practices.

#### Meaningfulness and spiritual significance of the experience

At 1-month follow-up, participants were asked to rate the personal meaningfulness and the spiritual significance of their study experience as well as subsequent changes in well-being or life satisfaction after the study retreat (Griffiths et al., 2006). The first item “How personally meaningful was the experience?” was rated from 0 to 7 (with 0 = no more than routine, everyday experiences; 6 = among the five most meaningful experiences of my life; and 7 = the single most meaningful experience of my life). The second item asked the participants to rate the degree to which the experience was spiritually significant to them (rated from 0 to 5, with 0 = not at all; 4 = among the five most spiritually significant experiences of my life; and 5 = the single most spiritually significant experience of my life). The third item asked the participants to rate whether the experience and the contemplation of it had led them to changes in their current sense of well-being or life satisfaction (rated from 0 to 6, 0 = decreased very much; 3 = no change; and 6 = increased very much).

#### Blinding efficacy and reaction to placebo

At the end of day 2 (i.e., the day of DMT-harmine or placebo administration), participants were asked to guess whether they had received DMT-harmine or placebo, how confident they felt regarding this assessment, and how satisfied or disappointed they felt about their respective study condition. The items regarding their confidence, satisfaction, and disappointment were rated on a Likert scale ranging from 0 (“not at all”) to 10 (“very much”). Taken together, these items were used to evaluate the blinding efficacy of the placebo and, in case of breaking blind, to assess participants’ satisfaction or disappointment about receiving DMT-harmine or placebo.

On the morning of day 2 before the DMT-harmine/placebo administration, participants’ expectations were assessed. Specifically, participants were asked to rate to which extent they expected a positive experience, challenging experience, transformative experience, meditative experience, or insightful experience, for the scenario of receiving DMT-harmine and separately also for the scenario of receiving the placebo.

### Data analysis

A two-way mixed ANOVA with a group (DMT-harmine vs placebo) as a between-subject factor and time (days 1–3) as a within-subject factor was used to assess the scores of TMS-13, PIS-6, EBI, MEQ30, and NADA-S throughout the retreat. A two-way mixed ANOVA with a group (DMT-harmine vs placebo) as a between-subject factor and time (baseline, post-retreat, 1-week follow-up) as a within-subject factor was used to assess FMI-13 scores. The same two-way mixed ANOVA but with one more measurement time point (baseline, day 2, post-retreat, and 1-week follow-up) as a within-subject factor was used to assess changes in scores on the SOCS-O and SOCS-S.

Post hoc pairwise comparisons were conducted using Wilcoxon signed-rank tests for comparisons between two time points and Mann–Whitney U tests for comparisons between the two groups. The significance level was set to α = 0.05 and adjusted for multiple testing using Bonferroni corrections in post hoc analyses.

To compare the DMT-harmine group to the placebo group in terms of their ratings related to meaningfulness and spiritual significance, blinding efficacy, and expectations, independent sample *t*-tests were used. If the requirements for independent sample *t*-tests were not given, Mann–Whitney *U* tests were computed. Pearson’s correlation coefficients were used to quantify the correlations between selected outcome variables. The data analysis was performed with R Studio version 2023.09.1+494 (R Core Team, 2023).

## Results

### Mindfulness

**Figure 1.**
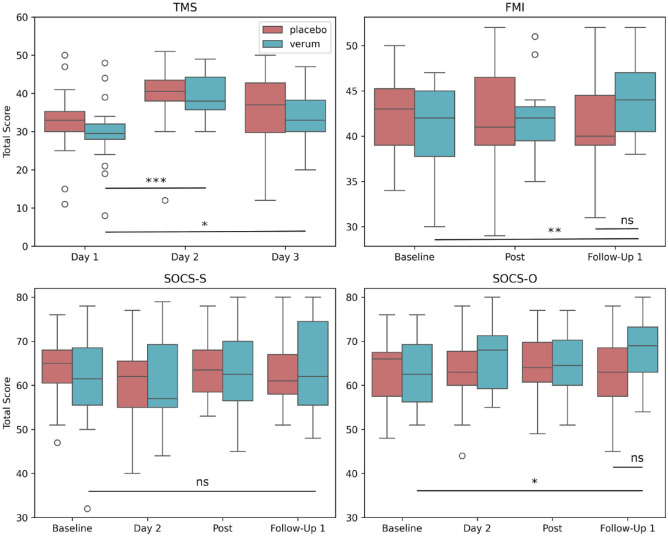
Boxplots of state mindfulness (Toronto Mindfulness Scale), trait mindfulness (Freiburg Mindfulness Inventory), self-compassion, and compassion for others (Sussex-Oxford Compassion Scales) over various time points. TMS: Toronto mindfulness scale; FMI: Freiburg mindfulness inventory; SOCS-S: Sussex-Oxford compassion for the self-scale; SOCS-O: Sussex-Oxford compassion for others scale. Outliers are indicated with circles. Asterisks indicate a significant difference compared to either the placebo group or another time point within the DMT-harmine group (*p < 0.05, **p < 0.01, ***p < 0.001, ns: nonsignificant). Regarding TMS scores, there was no significant effect of the interaction between the study group and time. While there was a significant interaction effect, there was no significant difference in FMI scores between the placebo group and the DMT-harmine group at baseline, 1 day after the retreat, or 1 week later. For both the SOCS-S and the SOCS-O, there was no significant effect of the interaction between the study group and time.

On each of the three retreat days, state mindfulness was assessed through the Toronto Mindfulness Scale (TMS-13). DMT-harmine did not significantly increase state mindfulness to a greater extent than placebo: No significant main effect of the study condition (DMT-harmine vs placebo) on the overall TMS score (*F* (1, 38) = 0.45, *p* = 0.505) nor of the interaction effect between the study condition and time point (day 1, day 2, and day 3) was found (*F* (2, 76) = 0.74, *p* = 0.481). However, time as a main effect was significant (*F* (2, 76) = 32.76, *p* < 0.001, η_p_^2^ = 0.46). Post hoc analyses using a Bonferroni correction revealed an increase in the overall TMS score in the DMT-harmine group from day 1 to day 2 (*p* < 0.001, mean ± SEM: 29.75 ± 1.89; mean ± SEM: 39.40 ± 1.32) and from day 1 to day 3 (mean ± SEM: 29.75 ± 1.89; mean ± SEM: 33.75 ± 1.63; *p* = 0.022; Figure 1 and Table 2).

**Table 2.** Participant ratings of state mindfulness, insight, and transcendence on day 1, day 2, and day 3 of the 3-day meditation retreat.

Measure	Placebo (*N* = 20)			DMT-harmine (*N* = 20)			Interaction effect (group * day)		
	Day 1, *M* (SEM)	Day 2, *M* (SEM)	Day 3, *M* (SEM)	Day 1, *M* (SEM)	Day 2, *M* (SEM)	Day 3, *M* (SEM)	*F*	*p*	η_p_^2^
**TMS**	32.30 (1.98)	39.50 (1.87)	35.65 (2.03)	29.75 (1.89)	39.40 (1.32)	33.75 (1.64)	0.74	0.481	0.02
**PIS**	26.45 (4.65)	36.33 (6.52)	38.37 (5.96)	15.98 (4.17)	41.93 (4.69)	52.65 (5.21)	5.70	0.005	0.13
**EBI**	23.03 (5.11)	27.33 (5.90)	27.26 (4.06)	15.14 (2.63)	47.85 (3.57)	39.26 (5.73)	10.04	<0.001	0.21
**MEQ**	1.10 (0.16)	1.71 (0.24)	1.49 (0.16)	1.18 (0.13)	3.23 (0.18)	1.76 (0.22)	16.19	<0.001	0.30
**NADA-S**	2.85 (0.44)	4.00 (0.60)	2.80 (0.43)	3.20 (0.47)	6.98 (0.42)	4.00 (0.54)	5.48	0.006	0.13

*N*: sample size; *M*: mean; SEM: standard error of the mean; TMS: Toronto mindfulness scale; PIS: psychological insight scale; EBI: emotional breakthrough inventory; MEQ: mystical experience questionnaire; NADA-S: nondual awareness dimensional assessment-state.

Using the Freiburg Mindfulness Inventory (FMI-13), trait mindfulness was assessed 1 day before and 1 day after the retreat, as well as at 1-week follow-up. We found a significant interaction effect between the study condition (DMT-harmine vs. placebo) and time (baseline, post-retreat, 1-week follow-up) for the overall FMI score (*F* (2, 70) = 7.26, *p* = 0.001, η_p_^20^ = 0.17) and a significant main effect of time (*F* (2, 70) = 4.22, *p* = 0.019, η_p_^2^ = 0.11). Post hoc analyses showed that at 1-week follow-up FMI scores in the DMT-harmine group were significantly higher than at baseline (*p* = 0.001) but not significantly higher than in the placebo group at 1-week follow-up (*p* = 0.076; Figure 1 and Table 3). Moreover, within the DMT-harmine group, trait mindfulness (FMI) at 1-week follow-up was moderately correlated with mystical experience scores (MEQ; *r* = 0.47; *p* < 0.001) and with state mindfulness (TMS; *r* = 0.55; *p* = 0.002) on the day of substance administration.

**Table 3.** Participant ratings of trait mindfulness, self-compassion, and compassion for others before and after the 3-day meditation retreat.

Measure	Placebo (*N* = 20)			DMT-harmine (*N* = 20)			Interaction effect (group * day)		
	Baseline, *M* (SEM)	Post, *M* (SEM)	1-week follow-up, *M* (SEM)	Baseline, *M* (SEM)	Post, *M* (SEM)	1-week follow-up, *M* (SEM)	*F*	*p*	η_p_^2^
**FMI**	42.00 (1.11)	42.25 (1.40)	41.42 (1.23)	41.05 (1.09)	42.25 (1.04)	44.00 (0.87)	7.26	0.001	0.17
**SOCS-S**	63.95 (1.77)	63.80 (1.65)	62.74 (1.74)	60.90 (2.32)	63.15 (2.10)	64.00 (2.62)	0.94	0.423	0.03
**SOCS-O**	63.55 (1.83)	64.80 (1.63)	63.11 (1.80)	62.60 (1.85)	65.25 (1.75)	67.38 (1.69)	2.33	0.079	0.06

*N*: sample size; *M*: mean; SEM: standard error of the mean; FMI: Freiburg mindfulness inventory; SOCS-S: Sussex-Oxford compassion for the self-scale; SOCS-O: Sussex-Oxford compassion for others scale.

### Compassion

Self-compassion and compassion for others were evaluated 1 day before the retreat, on day 2, 1 day after the retreat, and at 1-week follow-up, using the Sussex-Oxford-Compassion Scales (SOCS). Neither self-compassion nor compassion for others showed significant interaction effects between the groups and time points (*F* (3, 105) = 0.94, *p* = 0.423 for SOCS-S and *F* (3, 105) = 2.33, *p* = 0.079 for SOCS-O, Figure 1 and Table 3). Moreover, there was no significant main effect of the group (DMT-harmine vs placebo), neither in self-compassion (*F* (1, 35) = 0.06, *p* = 0.813) nor in compassion for others (*F* (1, 35) = 0.70, *p* = 0.408). However, while there was no significant main effect of time (baseline, day 2, post-retreat, 1-week follow-up) in compassion for others (*F* (3, 105) = 2.10, *p* = 0.105), self-compassion scores showed a significant effect of time (*F* (3, 105) = 3.64, *p* = 0.015, η_p_^2^ = 0.09). Within the DMT-harmine group, scores of self-compassion (SOCS-S) and compassion for others (SOCS-O) at 1-week follow-up were moderately correlated with scores of mystical experience (MEQ) (*r* = 0.58 for SOCS-S, *p* = 0.002; and *r* = 0.34 for SOCS-O, *p* = 0.039) and moderately to strongly with scores of state mindfulness (TMS) (*r* = 0.71 for SOCS-S, *p* < 0.001; and *r* = 0.68 for SOCS-O, *p* < 0.001) during the DMT-harmine experience.

### Insight

**Figure 2.**
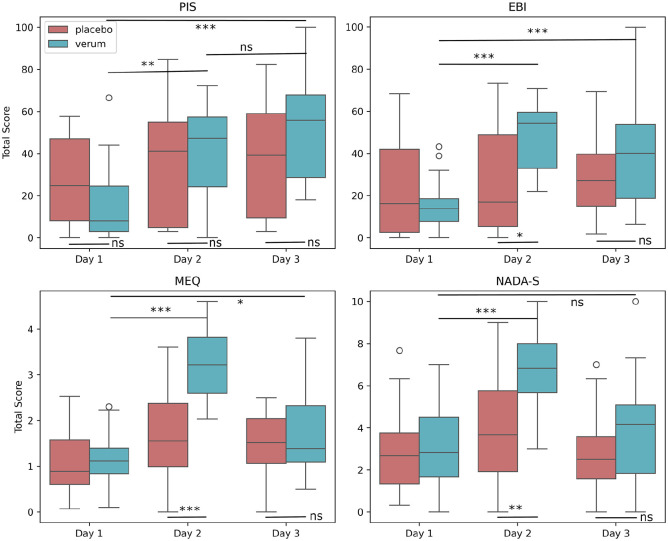
Levels of psychological insight (Psychological Insight Scale), emotional breakthrough (Emotional Breakthrough Inventory), mystical experience (Mystical Experience Questionnaire), and nondual awareness (Nondual Awareness Dimensional Assessment-State) on each day of the 3-day meditation retreat. PIS: psychological insight scale; EBI: emotional breakthrough inventory; MEQ: mystical experience questionnaire; NADA-S: nondual awareness dimensional assessment-state. Asterisks indicate a significant difference compared to either the placebo group or another time point within the DMT-harmine group (*p < 0.05, **p < 0.01, ***p < 0.001, ns: nonsignificant). The participants’ PIS scores showed a significant interaction effect between the group and time. EBI scores showed a significant interaction effect between group and time. EBI breakthrough scores were significantly higher in the DMT-harmine group than in the placebo group on day 2 but not on day 3. MEQ scores showed a significant interaction effect between group and time. On day 2, the MEQ score was significantly higher in the DMT-harmine group than in the placebo group (*p* < 0.001). There was a significant interaction effect between group and time on NADA-S ratings. On day 2, the NADA-S score was significantly higher in the DMT-harmine group than in the placebo group (*p* = 0.001).

Throughout the retreat, psychological insight was assessed on day 1, day 2, and day 3 by the participants, using the Psychological Insight Scale (PIS). There was no significant main effect of the group (DMT-harmine vs placebo). However, a significant effect was shown for a time as a main effect (*F* (2, 76) = 22.93, *p* < 0.001, η_p_^2^ = 0.38) and for the interaction effect between the group and time (day 1, day 2, day 3), *F* (2, 76) = 5.70, *p* = 0.005, η_p_^2^ = 0.13. Post hoc analyses revealed an increase in the overall PIS score in the DMT-harmine group from day 1 to day 2 (*p* = 0.001) and from day 1 to day 3 (*p* < 0.001) but not from day 2 to day 3 (*p* = 0.064). There were no significant effects for the pairwise comparisons between the DMT-harmine group and the placebo group for day 1, day 2, or day 3 (Figure 2 and Table 2). However, correcting for baseline differences between the two groups on day 1, an additional post hoc comparison of the deltas (day 2–day 1 and day 3–day 1) between the groups indicated that the DMT-harmine group reported a significantly larger increase in PIS scores from day 1 to day 3 compared to the placebo group (*p* = 0.001) but not from day 1 to day 2 (*p* = 0.072).

On the same days, also the level of emotional breakthrough was assessed, using the Emotional Breakthrough Inventory (EBI). Similarly to the PIS, there was no significant main effect of the group (DMT-harmine vs placebo) but a significant effect of time (*F* (2, 76) = 17.71, *p* < 0.001, η_p_^2^ = 0.32) and of the interaction between the group and time (day 1, day 2, day 3), *F* (2, 76) = 10.04, *p* < 0.001, η_p_^2^ = 0.21. Post hoc analyses revealed a significant increase in the EBI score in the DMT-harmine group from day 1 to day 2 (*p* < 0.001) and from day 1 to day 3 (*p* < 0.001) as well as a significantly higher EBI score in the DMT-harmine group compared to the placebo group on day 2 (*p* = 0.012) but not on day 3 (*p* = 0.126; Figure 2 and Table 2).

### Transcendence

On each day of the 3-day retreat, participants self-assessed the degree to which they had a mystical experience during that given day, using the MEQ. There was a significant main effect of group (DMT-harmine vs. placebo; *F* (1, 38) = 8.86, *p* = 0.005, η_p_^2^ = 0.19) and of time (days 1–3; *F* (2, 76) = 48.31, *p* < 0.001, η_p_^2^ = 0.56). Importantly, a significant interaction effect was found between group and time, *F* (2, 76) = 16.19, *p* < 0.001, η_p_^2^ = 0.30. Post hoc analyses revealed that the DMT-harmine group showed significantly higher MEQ scores on day 2 than on day 1 (*p* < 0.001) and day 3 than on day 1 (*p* = 0.011). Moreover, the DMT-harmine group showed higher MEQ scores than the placebo group on day 2 (*p* < 0.001) but not on day 3 (*p* = 0.705; Figure 2 and Table 2).

State non-dual awareness was also assessed daily during the retreat, using the Nondual Awareness Dimensional Assessment-State (NADA-S). Similarly to the MEQ, the NADA-S showed a significant main effect of group (DMT-harmine vs. placebo; *F* (1, 38) = 8.78, *p* = 0.005, η_p_^2^ = 0.19) and of time (day 1–3; *F* (2, 76) = 21.47, *p* < 0.001, η_p_^2^ = 0.36). Moreover, a significant interaction effect was found between group and time, *F* (2, 76) = 5.48, *p* = 0.006, η_p_^2^ = 0.13 (Figure 2 and Table 2). Post hoc analyses indicated that the DMT-harmine group showed significant increases in NADA-S scores from day 1 to day 2 (*p* < 0.001) but not from day 1 to day 3 (*p* = 0.235). Moreover, the DMT-harmine group showed significantly higher NADA-S scores compared to the placebo group on day 2 (*p* = 0.001) but not on day 3 (*p* = 0.126).

### Meaningfulness of the experience

**Figure 3.**
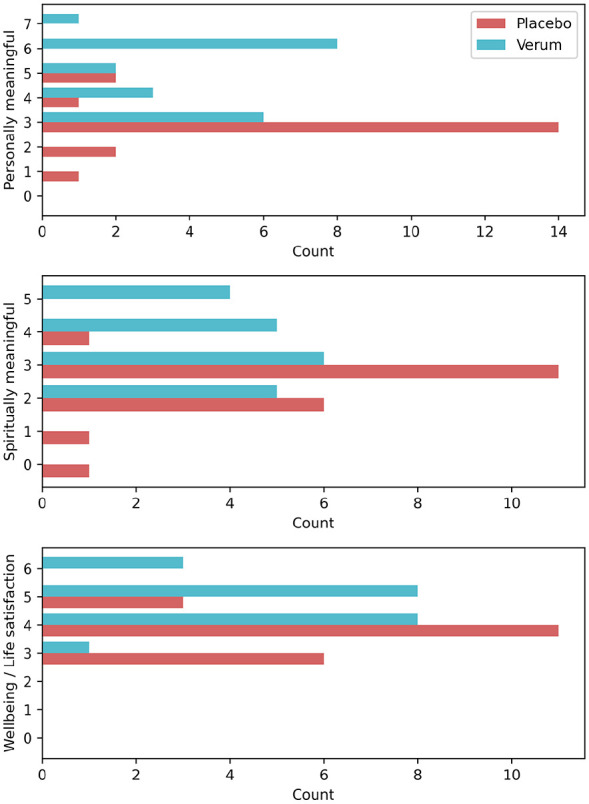
Participant ratings of meaningfulness, spiritual significance, and changes in well-being at 1-month follow-up. Bars show the total number of participants who rated each of the response options. The first panel displays the participant ratings on the first item “How personally meaningful was the experience?” (with 0 = no more than routine, everyday experiences; 6 = among the five most meaningful experiences of my life; and 7 = the single most meaningful experience of my life). The second panel displays the participant ratings of the degree to which the experience was spiritually significant to them (rated from 0 to 5, with 0 = not at all; 4 = among the five most spiritually significant experiences of my life; and 5 = the single most spiritually significant experience of my life). The third panel displays the participant ratings of the extent to which the experience and the contemplation of it have led them to changes in their current sense of well-being or life satisfaction (rated from 0 to 6, 0 = decreased very much; 3 = no change; and 6 = increased very much).

One month after the study retreat, participants were asked to rate the personal meaningfulness and spiritual significance of their experience and to assess to which degree it had led to changes in their current sense of well-being or life satisfaction. Participants in the DMT-harmine group rated their experience as significantly more personally meaningful, more spiritually significant, and more impactful toward increased well-being and life satisfaction (Figure 3 and Table 4).

**Table 4.** Personal meaningfulness, spiritual significance, and changes in well-being at 1-month follow-up.

Measure	Placebo	DMT-harmine
**How personally meaningful was the experience?**		
Mean (min/max score = 0–7)	3.05 (0.20)	4.75 (0.32)***
% rating top 5 most personally meaningful	0%	45%
% rating the single most personally meaningful	0%	5%
**How spiritually significant was the experience?**		
Mean (min/max score = 0–5)	2.5 (0.20)	3.4 (0.24)**
% rating top 5 most spiritually significant	5%	45%
% rating the single most spiritually significant	0%	20%
**Did the experience change your sense of well-being or life satisfaction?**		
Mean (min/max score = 0–6)	3.85 (0.15)	4.65 (0.18)**
% rating much increased or very much increased	15%	55%

Mean raw scores are shown with 1 SEM in parentheses where asterisks indicate a significant difference from the placebo group.

* *p* < 0.05, **p < 0.01, ***p < 0.001.

### Blinding efficacy

At the end of day 2, that is, the day of placebo or DMT-harmine administration, the participants were asked to guess whether they got placebo or DMT-harmine. 82.5% of participants guessed their group correctly (75% of the placebo group participants, and 90% of the DMT-harmine group participants).

Participants also rated their confidence about their guess (from 0 to 10; 0 = not at all; 10 = very much): While the overall assessed confidence was relatively high (mean ± SEM: 8.1 ± 0.43), there was no significant difference in confidence between the DMT-harmine group (mean ± SEM: 8.95 ± 0.39) and the placebo group (mean ± SEM: 7.25 ± 0.61), *z* = 1.7, *p* = 0.08. Moreover, those participants who guessed their group assignment correctly (whether in the placebo or DMT-harmine group) tended to be more confident in their assessment (mean ± SEM: 8.70 ± 0.38) than those who guessed incorrectly (mean ± SEM: 5.29 ± 1.23).

In addition, participants rated their levels of satisfaction and disappointment with their study condition (from 0 to 10; 0 = not at all; 10 = very much). While the overall self-rated satisfaction with the randomized assignment to the study condition was relatively high (mean ± SEM: 7.78 ± 0.45), the DMT-harmine group (mean ± SEM: 9.20 ± 0.39) was significantly more satisfied with their study condition than the placebo group (mean ± SEM: 6.35 ± 0.69), *z* = 3.7, *p* < 0.001. Accordingly, within a relatively low level of disappointment (mean ± SEM: 2.30 ± 0.53), the placebo group (mean ± SEM: 3.75 ± 0.78) was significantly more disappointed with their study condition than the DMT-harmine group (mean ± SEM: 0.85 ± 0.52) *z* = –3.1, *p* < 0.001.

Before the administration of DMT-harmine or placebo, we asked the participants to rate their expectations toward a positive, transformative, and insightful experience (from 0 to 4; 0 = not at all; 4 = very much), for DMT-harmine and placebo separately. The participants had higher expectations about DMT-harmine (mean ± SEM: 2.06 ± 0.13) than about placebo (mean ± SEM: 1.56 ± 0.11), *t*(78) = 2.91, *p* = 0.005.

## Discussion

The present randomized placebo-controlled study investigated the self-reported effects on mindfulness, compassion, insight, and transcendence of DMT-harmine-enhanced meditation compared to meditation with a placebo during a 3-day mindfulness retreat. We found that the most profound changes occurred in scores of insight (PIS, EBI) and transcendence (MEQ, NADA-S) as well as meaningfulness while mindfulness (TMS, FMI) and compassion (SOCS-S, SOCS-O) were changed only slightly or not significantly, compared to placebo.

Contributing to a rapidly growing body of research targeting potential synergistic effects between meditation and psychedelics, this present study brings further nuance into how psychedelics may support meditation practice and well-being. It builds on previous research that has classified meditation practices into different types (Dahl et al., 2015) which are in varying degrees related to core dimensions of well-being, namely awareness, compassion, insight, and purpose (Dahl et al., 2020). Relating to this framework, the results of the present study indicate that the psychedelic substance DMT combined with the MAO inhibitor harmine may support meditative processes, in particular through occasioning deeply meaningful mystical-type experiences with increased non-dual awareness and emotional breakthroughs that may facilitate subsequent meditative psychological insights. Within the four core dimensions of well-being, as proposed by Dahl et al. (2020), these results suggest that DMT-harmine may promote meditation-related well-being through eliciting experiences of insight, transcendence, and meaning rather than through mindfulness or compassion.

### Insight, transcendence, and meaning

Consistent with previous evidence that psilocybin-enhanced meditation, compared to meditation with a placebo, elicits greater scores on the 5D-ASC subscale of insightfulness (Smigielski et al., 2019a), also DMT-harmine-enhanced meditation elicited significantly greater increases in insight from day 1 to day 3 than meditation with a placebo. Moreover, our finding is consistent with previous literature on psychological insights related to psychedelic experiences (Aicher et al., 2024; Davis et al., 2021; Gandy et al., 2022; Peill et al., 2022). Being closely related to insight and consistent with the previous evidence of emotional breakthrough experiences occasioned by a variety of psychedelics (Roseman et al., 2019), including DMT-harmine (Aicher et al., 2024), our results represent a direct demonstration of a significantly greater increase in emotional breakthrough in meditation combined with DMT-harmine in comparison to meditation with a placebo.

Importantly, the present study’s finding of a DMT-harmine-occasioned increase in mystical experiences and non-dual awareness is consistent with previous psychedelics research on psilocybin in the context of spiritual or meditative practice (Griffiths et al., 2018; Smigielski et al., 2019a) and in other not explicitly meditative contexts including psilocybin (Griffiths et al., 2016, 2006, 2008, 2011; Garcia-Romeu et al., 2015) and ayahuasca or ayahuasca-analogs (Palhano-Fontes et al., 2019; Pontual et al., 2022; Sarris et al., 2021). Psychedelics supposedly promote mystical experiences and non-dual awareness by attenuating self-boundaries and changes in brain network function underlying self-perception (Smigielski et al., 2019b). Such profound experiences can result in lasting adaptations in attitudes and behaviors, thereby supporting the beneficial outcomes of psychedelic-assisted therapy (Scheidegger, 2021).

Regarding the meaningfulness of the experience, the DMT-harmine group rated their study experience as significantly more meaningful and spiritually significant than the placebo group. Our findings highlight that DMT-harmine combined with meditation can occasion highly meaningful and spiritually significant experiences, supporting a self-attributed sense of well-being. The ratings from the present study (personal meaningfulness: 45% among the top 5) are slightly higher yet relatively consistent with Smigielski et al.’s (2019a) previous findings on psilocybin in a similar meditative context (35% among the top 5) and considerably higher than Aicher and Wicki et al.’s (personal communication) results on DMT-harmine in healthy non-meditating subjects in a laboratory context (16% among the top 5). However, even higher personal significance ratings (84% among the top 5) were reported in a previous study by Griffiths et al. (2018) on psilocybin combined with spiritual support, which illustrates that contextual and cultural differences can play an important role in shaping the interpretation of psychedelic experiences (Hartogsohn, 2016, 2017; Meling and Scheidegger, 2023, 2024). A similar pattern is found for the ratings of spiritual significance (among the top 5; 45% in the present study; 96% in Griffiths et al., 2018; data not available in Smigielski et al., 2019a; 12% in Aicher and Wicki et al., personal communication) and increases in well-being or life satisfaction (much increased or very much increased; 55% in the present study; 92% in Griffiths et al., 2018; data not available in Smigielski et al., 2019a; 12% in Aicher and Wicki et al., personal communication).

### Mindfulness and compassion

While previous research has suggested the enhancement of mindfulness-related capabilities after intake of psilocybin (Smigielski et al., 2019a) and ayahuasca (Sampedro et al., 2017; Soler et al., 2016), the present study did not find a direct increase in self-reported mindfulness after intake of DMT-harmine in comparison to placebo in the context of a meditation retreat.

While our finding of a short-term increase in state mindfulness associated with a psychedelic substance is consistent with the previous literature on psilocybin-enhanced meditation (Smigielski et al., 2019a), we found a stronger *placebo*-related increase in state mindfulness than in the previous literature, speaking for a relevant impact of the overall meditation setting. Moreover, while a cross-sectional survey has not found any linkages between lifetime use of classic psychedelics and the mindfulness-related capability of decentering in a representative sample of American adults (Simonsson and Goldberg, 2023), previous open-label, non-placebo-controlled, ayahuasca studies have shown pre-post increases in the mindfulness-related capabilities of decentering, nonjudgment, and non-reactivity 24-h after ingesting ayahuasca (Sampedro et al., 2017; Soler et al., 2016). Interestingly, this increase in mindfulness after ayahuasca was indeed replicated in the present study with DMT-harmine as we found a significant increase in mindfulness, including its two subdimensions of curiosity and decentering, from day 1 to day 2 in the DMT-harmine group. More importantly, however, the present study showed a very similar state mindfulness increase in the placebo group, rendering no significant difference to the DMT-harmine group and thereby no significant interaction effect between group and time.

In contrast to a previous study that showed increases in trait mindfulness 1 day after a 5-day psilocybin-enhanced meditation retreat (Smigielski et al., 2019a), the present study with DMT-harmine did not find increased trait mindfulness 1 day after the 3-day retreat but found a significantly stronger within-group increase 1 week later. Potentially, this may be due to differences regarding the specific drug effects (psilocybin vs DMT-harmine) or dose strength, the length of the retreat, or other causal factors regarding set and setting. In addition, the poly-pharmacology of harmine, including its effects on various CNS receptors, could also contribute to differences in drug effects.

Compassion is central to the second of four core dimensions of well-being by Dahl et al. (2020), we explored the potential impact of DMT-harmine on compassion and found no supporting evidence of DMT-harmine-enhanced compassion for oneself (*p* = 0.423) or for others (*p* = 0.079) in comparison to placebo. This finding of the present study contrasts a previous study showing a significant pre-post increase in compassion for oneself and for others 24 h after a DMT-harmine session in healthy non-meditating participants (Aicher and Wicki et al., personal communication). However, both studies found no sustained effects at follow-up time points after a single administration.

While Payne et al. (2021) have argued for the hypothesis that psychedelics may occasion a type of mindfulness that is kinder and more compassionate, the present study is one of the first reported attempts to test the impact of psychedelic-enhanced meditation on compassion and shows no direct increase in self-compassion or compassion for others greater than placebo combined with meditation. Demonstrating the effects of a single psychedelic administration on compassion can be challenging due to the complex and multifaceted nature of this construct, the variability of psychedelic experiences among individuals, and the necessity of an integration process to embody potential increases in compassion toward self and others, which was not provided in our studies.

### Benefits and risks of combining psychedelics and meditation

These results from the present study imply that DMT-harmine may support meditation and meditation-related well-being in particular through eliciting experiences of insight, transcendence, and meaning rather than through enhancement of mindfulness or compassion. This implies potential benefits and risks (Meling and Ehrenkranz et al., 2024).

In an ecological notion of meditation (Vervaeke, 2022), various types of practices complement each other toward a more holistic sense of well-being. Likewise, a crucial claim from many discourses in the Buddhist tradition is that wisdom and compassion must be balanced to contribute to well-being and flourishing: The risk of wisdom (i.e., transcendent insight) without compassion is that it may lead to apathy and dismissal of “worldly” activities while compassion without wisdom may lead to suffering when others’ aspirations do not correspond to those one has imagined in one’s mind (Condon et al., 2019).

Attaining mystical experiences or non-dual awareness is a process that unfolds over time, through consistent meditation practice, introspection, and a commitment to growth and self-discovery. On this path, practitioners with a solid practice of mindfulness and compassion may benefit from pharmacologically augmented altered states of consciousness such as an enhanced sense of insight, transcendence, and meaningfulness. But an important potential risk is that overly self-transcendent-oriented practices with psychedelic drugs may lead to the aforementioned danger of “wisdom without compassion,” if not properly balanced out with a firm grounding in mindfulness and compassion practice.

### Implications for further research

Future research on the nuances of potential synergistic effects between psychedelics and meditation may address the following questions and hypotheses: Are there differential effects in response patterns to psychedelics between meditators who score high on mindfulness and compassion but low on insight measures versus meditators who score high on insight but low on mindfulness and compassion? Also, further research is needed to extrapolate these findings from DMT-harmine to other combinations between various styles of meditation (compassion, insight, mindfulness, etc.) and various psychedelic substances and dosages (high dose vs. low dose of LSD, mescaline, 5-MeO-DMT, etc.). Moreover, while the present study investigated the combined effects of DMT and harmine, further research should consider differentiating the effects of harmine alone to provide insights into the pharmacological contributions of the DMT-harmine combination versus harmine alone, which has multiple CNS activities. Notably, the incremental sublingual doses of harmine administered in this trial were lower than in an average oral formulation of ayahuasca that may contain a broad ß-carboline (including harmaline and THH) concentration range of a couple of hundred milligrams (Egger et al., 2024). It is possible that using a different regimen with varying dosing frequencies and ratios of DMT to harmine could lead to different experiential outcomes. Although the DMT-harmine combination significantly increased MEQ and NADA-S scores compared to placebo, studies with high doses of psilocybin or traditional ayahuasca have reported more intense mystical experiences (Griffiths et al., 2018; Palhano-Fontes et al., 2019; Smigielski et al., 2019a). The lower harmine (resp. ß-carboline) content and the intermittent sublingual administration method, which were specifically designed for compatibility with meditative practice, likely resulted in a significant but less intense experience profile. Future studies could explore different dosing regimens and administration methods to optimize the mystical experience potential of DMT-harmine combinations. Future research may also investigate which practitioners may benefit most from psychedelic interventions (novices vs intermediate vs expert meditators), potentially also including qualitative interviews on the perceived benefits and challenges. Taken together, further research is required to reveal further potential risks and particular benefits of meditation combined with psychedelics.

### Limitations

A common major limitation in psychedelic research studies is the breaking blind problem (Van Elk and Fried, 2023). Assessing and reporting the blinding efficacy, we have shown that most of the participants (82%) guessed correctly whether they had received a placebo or DMT-harmine and that they were relatively confident about their guess, likely due to the distinct experiential quality of the perceptible effects of psychedelic drugs. Moreover, before the experience, the participants had rated significantly higher expectations for DMT-harmine than for placebo while after the experience the placebo group reported significantly higher levels of disappointment with their study condition than the DMT-harmine group. While the use of an inactive placebo was motivated by establishing a clear baseline to allow for the measurement of the absolute effects of DMT-harmine through minimizing confounding variables, the different expectations and the high rate of participants breaking blind represent an important limitation of the present study. Another major limitation of this study is the absence of a control group for meditation. Without a non-meditation control condition, we cannot determine the specific effects of DMT-harmine alone. Future studies should consider a 2 × 2 design with meditation versus no-meditation and DMT-harmine versus placebo to assess interactions comprehensively. Similarly, the absence of a harmine-only condition limits an understanding of its independent effects, particularly in combination with meditation. Future studies may benefit from including both harmine alone and harmine with meditation conditions to further elucidate their distinct and interactive influences on experiential outcomes. Moreover, given the variety of meditation practices, their differential effects, and the variety of meditation traditions or meditation styles in which the participants were primarily practicing, it remains unclear whether DMT-harmine was combined with the same kind of meditation practice in each participant. Another limitation concerns the absence of physiological or behavioral measures, such as assessments of attentional blink, emotional regulation, social behavior, and implicit bias. A final limitation concerns the homogeneity of the study sample (mostly college-educated and White), calling into question the generalizability of this study’s results to a broader diversity of populations. Future research in more diverse samples is required.

## Conclusion

While research into the intersection of meditation and psychedelics has gained momentum, the present article presents the first trial to investigate the effects of a substance other than psilocybin during meditation. The combined effects of meditation with an ayahuasca-analogue sublingual formulation of DMT and harmine on measures of (1) mindfulness, (2) compassion, (3) insight, and (4) transcendence were investigated in 40 intermediate meditation practitioners during a 3-day meditation group retreat. We showed that meditators who received DMT-harmine reported greater levels of mystical-type experiences, non-dual awareness, and emotional breakthrough during the acute substance effects than meditators who received placebo. While psychological insight increased throughout the retreat, the DMT-harmine group showed, if corrected for baseline difference, greater psychological insight 1 day after substance administration than the placebo group. Across time points, state mindfulness, trait mindfulness, self-compassion, and other-oriented compassion were not significantly different between the DMT-harmine group and the placebo group. At 1-month follow-up, the DMT-harmine group rated their experience as significantly more personally meaningful, spiritually significant, and well-being-enhancing than the placebo group. This study provides novel evidence supporting the notion that the combination of the psychedelic compound DMT with harmine has the potential to enhance meditation through an increased sense of insight, transcendence, and meaning, offering valuable insights into the intersection of psychedelics and meditative practices.

## Supplemental Material

sj-docx-1-jop-10.1177_02698811241282637 – Supplemental material for Meditating on psychedelics. A randomized placebo-controlled study of DMT and harmine in a mindfulness retreat Supplemental material, sj-docx-1-jop-10.1177_02698811241282637 for Meditating on psychedelics. A randomized placebo-controlled study of DMT and harmine in a mindfulness retreat by Daniel Meling, Klemens Egger, Helena D Aicher, Javier Jareño Redondo, Jovin Mueller, Joëllle Dornbierer, Elijah Temperli, Emilia A Vasella, Luzia Caflisch, David J Pfeiffer, Jonas TT Schlomberg, John W Smallridge, Dario A Dornbierer and Milan Scheidegger in Journal of Psychopharmacology
