# Supplementary material for: Meditating on psychedelics. A randomized placebo-controlled study of DMT and harmine in a mindfulness retreat
Source: J Psychopharmacol. 2024 Sep 27;38(10):897–910. doi: 10.1177/02698811241282637 (PMC11487865; doi:10.1177/02698811241282637)
Supplement: sj-docx-1-jop-10.1177_02698811241282637 – Supplemental material for Meditating on psychedelics. A randomized placebo-controlled study of DMT and harmine in a mindfulness retreat [file sj-docx-1-jop-10.1177_02698811241282637.docx]

**Supplemental Material**

Meling, D., Egger, K., Aicher, H.D., Jareño Redondo, J., Mueller, J., Dornbierer, J., Temperli, E., Vasella, E.A., Caflisch, L., Pfeiffer, D.J., Schlomberg, J.T.T., Smallridge, J.W., Dornbierer, D.A., Scheidegger, M. Meditating on psychedelics. A randomized placebo-controlled study of DMT and harmine in a mindfulness retreat

This supplemental material has been provided by the authors to give readers additional

information about the study.

**Table S1. Participant Characteristics in Comparison Between the DMT-harmine group and Placebo group.**

|  | DMT-harmine group | Placebo group | *p* | SMD |
| --- | --- | --- | --- | --- |
| n | 20 | 20 |  |  |
| Age (mean (SD)) | 41.85 (11.66) | 45.60 (8.38) | 0.250 | 0.369 |
| Sex (%) |  |  |  |  |
| male | 11 (55.0) | 11 (55.0) | 1.000 | <0.001 |
| female | 9 (45.0) | 9 (45.0) |  |  |
| Hours of meditation practice (mean (SD)) | 2125.00 (1509.49) | 2720.00 (2182.37) | 0.322 | 0.317 |
| Highest Education level (%) |  |  |  |  |
| primary school degree | 2 (10.0) | 1 (5.0) | 0.669 | 0.501 |
| secondary school degree | 0 (0.0) | 1 (5.0) |  |  |
| high school degree | 1 (5.0) | 1 (5.0) |  |  |
| university degree | 16 (80.0) | 17 (85.0) |  |  |
| other school degree | 1 (5.0) | 0 (0.0) |  |  |
| Years of education (mean (SD)) | 17.90 (5.32) | 18.35 (5.34) | 0.791 | 0.084 |
| Ethnicity (%) |  |  |  |  |
| White | 19 (95.0) | 19 (95.0) | 1.000 | <0.001 |
| Hispanic | 1 (5.0) | 1 (5.0) |  |  |
| Religious belief (%) |  |  |  |  |
| Christian | 4 (20.0) | 3 (15.0) | 0.691 | 0.657 |
| Buddhist | 5 (25.0) | 3 (15.0) |  |  |
| Atheist | 0 (0.0) | 1 (5.0) |  |  |
| Pantheist/Animist | 0 (0.0) | 1 (5.0) |  |  |
| Spiritual, but not religious | 7 (35.0) | 9 (45.0) |  |  |
| None | 3 (15.0) | 3 (15.0) |  |  |
| Other | 1 (5.0) | 0 (0.0) |  |  |

Values are either given as mean (standard deviation) or as frequency (%) including standard mean difference (SMD) and *p-*value.
